# Niche Differentiation Characteristics of Phytoplankton Functional Groups in Arid Regions of Northwest China Based on Machine Learning

**DOI:** 10.3390/biology14111564

**Published:** 2025-11-07

**Authors:** Long Yun, Fangze Zi, Xuelian Qiu, Qi Liu, Jiaqi Zhang, Liting Yang, Yong Song, Shengao Chen

**Affiliations:** 1 Tarim Research Center of Rare Fishes, State Key Laboratory Incubation Base for Conservation and Utilization of Bio-Resource in Tarim Basin, College of Life Sciences and Technology, Tarim University, Alar 843300, China; 10757232147@stumail.taru.edu.cn (L.Y.); or 10757213076@stumail.taru.edu.cn (F.Z.); 10757231124@stumail.taru.edu.cn (X.Q.); 10757241105@stumail.taru.edu.cn (Q.L.); 10757242168@stumail.taru.edu.cn (J.Z.); 10757231127@stumail.taru.edu.cn (L.Y.); 2 College of Material Science and Engineering, Beijing University of Chemical Technology, Beijing 100029, China

**Keywords:** arid zones, phytoplankton, ecological niche, interspecific connectivity

## Abstract

**Simple Summary:**

To reveal the distribution patterns of phytoplankton in reservoir ecosystems, this study selected four representative reservoirs in the Tarim River Basin, Xinjiang, China. It systematically analysed the structural characteristics of phytoplankton communities and simultaneously clarified the correlation between these communities and various environmental factors and their interrelationships. From 2023 to 2024, seasonal sampling of phytoplankton functional groups was conducted in 4 reservoirs in the upper Tarim River Basin. Eighteen functional groups were identified (8 dominant ones), with pH and electrical conductivity as key driving factors. According to the results of Redundancy Analysis (RDA), on the RDA1 axis, pH value, electrical conductivity (COND), and total dissolved solids (TDS) showed positive correlations, while all other environmental factors exhibited negative correlations; on the RDA2 axis, dissolved oxygen (DO) and pH value presented positive correlations, and all other environmental factors displayed negative correlations. According to the analysis of interspecific association using the Association Coefficient (AC), among all the reservoirs studied, only Reservoir Shangyou Reservoir (SY) exhibited a higher number of negative correlations than positive correlations in the interrelationships of phytoplankton functional groups; the other reservoirs all showed a greater number of positive correlations than negative ones. Communities in three reservoirs, including Duolang Reservoir (DL), are characterised by positive associations (stable), whereas SY is characterised by negative associations (early successional stage). This reveals interspecific interactions and mechanisms, providing a basis for ecological management.

**Abstract:**

This study investigates the distribution patterns, interspecific relationships, and community stability mechanisms of phytoplankton functional groups, aiming to elucidate the ecological processes that drive phytoplankton communities in aquatic ecosystems of arid regions. We conducted seasonal sampling from 2023 to 2024 at four auxiliary reservoirs in the Tarim River Basin, namely Shangyou Reservoir (SY), Shengli Reservoir (SL), Duolang Reservoir (DL), and Xinjingzi Reservoir (XJZ). In recent years, researchers have grouped phytoplankton into functional groups based on their shared morphological, physiological, and ecological characteristics—with these three types of traits serving as the core criteria for distinguishing different functional groups. A total of 18 functional groups were identified from the phytoplankton collected across four seasons, among which eight (A, D, H1, L0, M, MP, P, and S1) are dominant. Redundancy Analysis (RDA) indicated that environmental factors such as pH, electrical conductivity (COND), and dissolved oxygen (DO) are key driving factors affecting phytoplankton functional groups. Interspecific association analysis showed that the phytoplankton communities in DL, SL, and XJZ reservoirs were dominated by positive associations, reflecting stable community structures that are less prone to drastic fluctuations under stable environmental conditions. In contrast, the SY Reservoir was dominated by negative associations, indicating that it is in the early stage of succession with an unstable community. This may be related to intense human disturbance to the reservoir and its role in replenishing the Tarim River, which leads to significant water level fluctuations. The results of the Chi-square test and Pearson correlation analysis showed consistent trends but also differences: constrained by the requirement for continuous normal distribution, Pearson correlation analysis identified more pairs of negative associations, reflecting its limitations in analysing clumped-distributed species. Random forest models further indicated that functional groups M, MP, L0, and S1 are the main positive drivers of interspecific relationships. Among them, the increase in S1 can promote the growth of functional groups dominated by *Navicula* sp. and *Chroococcus* sp. by reducing resource competition. Conversely, the expansion of functional group H1 inhibits other groups, which is related to its adaptive strategy of resisting photo-oxidation in eutrophic environments. This study reveals the patterns of interspecific interactions and stability mechanisms of phytoplankton functional groups in arid-region reservoirs, providing a scientific basis for the management and conservation of aquatic ecosystems in similar extreme environments.

## 1. Introduction

The arid region of Northwest China is one of the areas with extremely scarce water resources worldwide. Meanwhile, the efficiency of water resource utilisation in this region remains at a relatively high level. As a typical freshwater ecosystem in this region, the Tarim River Basin is located in the Tarim Basin surrounded by the Tianshan Mountains and the Kunlun Mountains, nurturing the longest inland river in China. Its unique water body structure system has formed against a geographical background characterised by sparse vegetation, scarce rainfall, and a fragile ecological environment. Such special habitat conditions have shaped the unique aquatic ecological characteristics of this river basin [1,2]. Although the Tarim River Basin is endowed with abundant groundwater resources, their spatial distribution is significantly uneven, and the water generally has a high degree of mineralisation, making exploitation and utilisation rather difficult. Against this backdrop, the construction of reservoir projects holds irreplaceable strategic significance for resource development and human survival security in the arid regions of Northwest China [3]. As a core facility for regional water resource storage and regulation, reservoirs hold a strategic position in water resource management in this region. Large surface water carriers, leveraging scientific water resource scheduling capabilities, demonstrate irreplaceable functional value across three key dimensions: supporting economic development, improving the ecological environment, and safeguarding basin ecological security. Currently, within the water resource pattern of the Tarim River Basin, SY, SL, DL, and XJZ have emerged as core hubs of large-scale freshwater surfaces in the region, holding strategic significance for basin-wide water resource allocation and ecological stability [4].

As a key primary producer in aquatic ecosystems, phytoplankton plays a crucial role in maintaining the stability and integrity of ecosystems [5]. Research on the mechanisms of phytoplankton community assembly has long been a core issue in studies on the maintenance mechanisms of aquatic biodiversity [6]. It serves as the theoretical basis for aquatic ecological protection. In recent years, researchers have grouped phytoplankton with similar morphological, physiological, and ecological characteristics into functional groups [7], and evaluated the health of aquatic ecosystems based on their species functionality [8]. Compared with traditional taxonomy, the analysis of functional group composition has more advantages in assessing the potential responses of phytoplankton to environmental changes [9]. Since the primary basis for dividing functional groups lies in the similarities of phytoplankton, including their morphological characteristics and habitat adaptability traits, this approach can not only simplify research on phytoplankton communities but also enable the study of aquatic ecosystems from the perspective of species functionality. Moreover, it can more accurately reveal the relationships between phytoplankton and environmental factors. Currently, this method has been widely applied in studies on lentic waters such as lakes and reservoirs [10,11]. Currently, research on the relationship between phytoplankton functional groups and the aquatic environment has been widely applied. In the Wujiang River-reservoir system, the correlation between nutrients and functional groups is relatively weak [12]. Water temperature, pH, dissolved oxygen, and turbidity are closely related to the composition of phytoplankton functional groups in the western Yangcheng Lake [13]. In Siming Lake Reservoir, water temperature, transparency, nitrate nitrogen, and zooplankton all affect the seasonal succession of phytoplankton functional groups [14]. The seasonal succession of phytoplankton functional groups in Dianshan Lake is influenced by water level fluctuations [15]. The findings from Max M. Tilzer’s research on Lake Tahoe—a high-altitude lake in the United States—demonstrated that phosphate limits the biomass of phytoplankton [16]. However, studies on the distribution patterns of phytoplankton functional groups in reservoirs in high-latitude, alpine, and arid regions remain scarce, and investigations into the interrelationships among these groups are also rarely conducted. It is generally believed that communities are constructed through the combined action of deterministic and stochastic processes. Deterministic processes refer to the influences of interspecific interactions within communities and environmental factors [17,18]. However, stochastic processes suggest that the community structure of plankton is also regulated by random factors such as limited dispersal and passive migration [19,20]. Therefore, quantifying determinism and stochasticity is crucial for exploring the mechanisms of community assembly.

Currently, there is no consensus on the assembly mechanisms of phytoplankton communities in large water bodies of the arid regions in Northwest China and their response relationships to changes in environmental factors. As a key entry point for revealing the aquatic ecological structure in the arid regions of Northwest China, researching the assembly mechanisms and dynamic changes in phytoplankton communities in the Tarim River Basin holds significant scientific importance. This study hypothesises that the assembly and evolution of phytoplankton in water bodies of the arid regions in Northwest China are mainly influenced by spatial heterogeneity, seasonal variations, environmental factors, and intra- and interspecific interactions among phytoplankton. The questions to be addressed in this study are as follows: the characteristics of phytoplankton community structure in reservoirs in arid regions; the functional group composition of phytoplankton communities during the wet season and dry season; the driving factors of phytoplankton community changes in reservoirs in arid regions; as well as the niche overlap, niche breadth, and interspecific associations among phytoplankton functional groups within the community. The top-down effects of zooplankton mediated by grazing and resource pre-emption. The most notable aspect lies in the interspecific interactions among different phytoplankton functional groups. This study aims to predict the niche of dominant functional groups within phytoplankton functional communities in the arid regions of Northwest China. It is expected to provide innovative insights into the ecological patterns and community assembly mechanisms of phytoplankton in large water surface ecosystems, as well as improve the aquatic ecological health assessment system. The research findings will lay a scientific foundation for the systematic monitoring of phytoplankton diversity in environmental assessment and the protective development of fishery resources in large water surface areas.

## 2. Materials and Methods

### 2.1. Study Area

**Figure 1:**
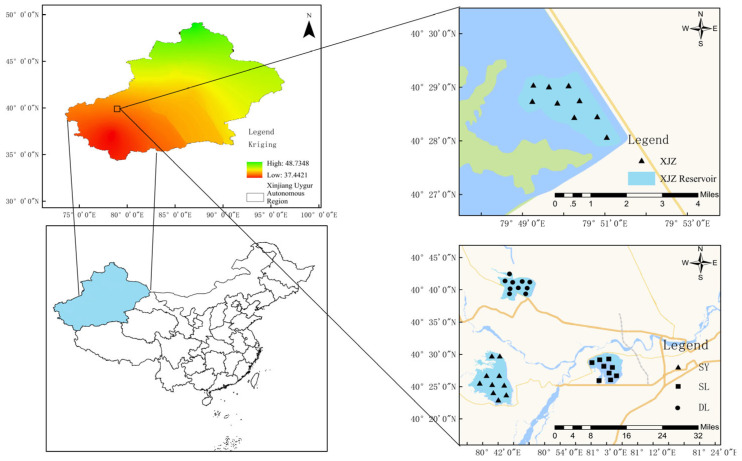
Phytoplankton sampling map of Tarim River valley reservoir.

The Tarim River Basin is located in the southern part of Xinjiang Uygur Autonomous Region, China, within the Tarim Basin, which is enclosed by the Tianshan Mountains and the Kunlun Mountains. With a main stream stretching 2179 km, it holds the title of China’s longest inland river [21]. From April 2023 to March 2024, 40 sampling points were established in the Tarim River Basin, covering its four key reservoirs: SY, SL, DL, and XJZ (Figure 1). The Tarim River Basin has distinct high-flow and low-flow seasons, with their time frames clearly defined. The high-flow season of the basin is concentrated in summer. This period sees abundant river water, primarily because rising temperatures significantly boost glacial meltwater, and even the minimal summer precipitation contributes to runoff—both factors together drive the high flow. In contrast, the low-flow season spans a longer period, specifically from late autumn to early spring of the next year. During this time, low temperatures in runoff-producing areas limit water sources, and the already scarce precipitation falls as solids (such as snow or ice), further reducing river discharge and maintaining the low-flow state., unable to directly generate runoff. Seasonal snow, glaciers, and permanent snow cover also cannot replenish the river due to low temperatures [22,23]. Samples were collected during all four seasons: spring (May), summer (July), autumn (October) and winter (November). Spring and summer represent the wet season, while autumn and winter correspond to the dry season.

### 2.2. Sample Collection and Determination Methods

#### 2.2.1. Physicochemical Indices of the Water

The WT (Water Temperature), DO (Dissolved Oxygen), pH, SAL (Salinity), EC (Electrical Conductivity), COND (Conductivity), and TDS (Total Dissolved Solids) data were measured in situ using a water quality detector (the ATI PQ45 multiparameter water quality analyzer from the United States, Beijing Zhongheng Rixin Technology Co., Ltd., Beijing, China). Water transparency was determined utilising a JCT-8S model Secchi disc, which is a product of JCT-8S Secchi disk from Qingdao Juchuang Environmental Protection Group Co., Ltd., Qingdao, China. A Global Positioning System (GPS), specifically the LS-GPS-639 model manufactured by GPS, the LS-GPS-639 from Qingdao Lvsheng Biotechnology Co., Ltd., Pingdu, China, was employed to record parameters like latitude, longitude, and altitude. Water chemical parameters were determined in the laboratory following the guidelines in the “Methods for the Examination of Water and Wastewater (Fourth Edition)” [24].

#### 2.2.2. Phytoplankton

A 5 L plexiglass water sampler (Germany, HYDRO-BIOS GmbH, Kiel, Germany) was used to collect 1.5 L of water samples at each sampling site for phytoplankton quantification. Lugol’s reagent was immediately added for fixation (with a final concentration of 1.5%). After being brought back to the laboratory and allowed to stand for 48 h, the supernatant was aspirated using the syphon method, and the sample was concentrated to 50 mL for identification [25,26]. Phytoplankton quantification was performed using microscopic examination and counting. Since the cell volume of 1 mm^3^ of phytoplankton is approximately equivalent to 1 mg of biomass (calculated as wet weight), the biomass (in mg·L^−1^) can be directly converted from the algal volume [25]. By calculating the abundance of phytoplankton functional groups at each sampling site from 2023 to 2024, functional groups with an abundance greater than 5% are defined as the dominant functional groups in the region [27].

The grid-line method in the “Determination of Phytoplankton in Water—0.1 mL Counting Chamber-Microscope Counting Method: the concentrated 30 mL water sample was shaken well, and 0.1 mL of the sample was pipetted into a 0.1 mL counting chamber using a 0.1 mL pipette. After covering with a coverslip (22 mm × 22 mm), counting was performed by species under a high-power microscope, targeting 30 small grids in the 2nd, 5th, and 8th rows of the counting chamber (the entire chamber has 10 rows, totalling 100 grids). The counting formula for phytoplankton in 1 L of water is as follows [25,26]:

$$N=\frac{10V_{0}}{3V_{1}}\times N_{1}$$

In the formula:

*N*: Number of phytoplankton per litre of water (ind./L);

*N*_1_: Counted number of phytoplankton(ind./L);

*V*_0_: The volume of the 1 L water sample after precipitation and concentration (mL);

*V*_1_: Volume of the counted sample (mL).

### 2.3. Division of Phytoplankton Functional Groups

Phytoplankton functional group (FG) classification was performed in accordance with the scheme put forward by Reynolds et al. in 2002 [7] and revised by Padisák et al. in 2009 [28], the phytoplankton collected across four seasons from the affiliated reservoirs of the Tarim River Basin can be categorised into 18 functional groups, namely A, D, E, F, H1, J, K, L0, M, MP, N, P, S1, SN, X1, X2, and Y. Among these, 8 groups—A, D, H1, L0, M, MP, P, and S1—are the dominant ones. The representative algal genera and habitat characteristics of each phytoplankton functional group in the affiliated reservoirs of the Tarim River Basin are presented in Table 1.

### 2.4. Data Processing and Analysis

#### 2.4.1. Calculation of Dominant Species

The calculation formula for the dominant species of phytoplankton is as follows [29]:

$$Y=((\frac{n_{i}}{N})f_{i})$$

In the formula:

*n_i_*: The variable represents the proportion of individuals belonging to the i-th species relative to the overall number of individuals;

*N*: The total number;

*f_i_*: The frequency of occurrence of the i-th species. When *Y* > 0.02, this species is regarded as a dominant species.

#### 2.4.2. Redundancy Analysis (RDA)

DCA analysis was carried out using Canoco for Windows 4.5 software, and the SD value was >3. Therefore, RDA was carried out on species data and related environmental factor data to investigate the effects of various environmental factors on zooplankton [30].

#### 2.4.3. Correlation Analysis and Mantel Test Correlation Test

After the Phytoplankton functional groups communities were processed by log10 to make them more normal, the average clustering method and Euclidean distance algorithm were used to conduct Pearson correlation analysis to explore the correlation between different Phytoplankton functional groups communities and environmental factors [31]. One-way ANOVA was used to analyse environmental factors with IBM SPSS Statistics 21 software to study the differences in environmental factors at different times, and *p* < 0.05 was used to indicate significant differences. In order to test the significance of environmental factors and Phytoplankton functional groups communities, Mantel test correlation was used, Bonferroni method was used for *p* value correction, The analysis result of Mantel test heat-map plot was generated using the R 4.3.1 software package “vegan”. The Variance Inflation Factor (VIF) was calculated to ensure that there is no collinearity among environmental variables. All the above analysis has been carried out Homogeneity test and normal distribution correction.

#### 2.4.4. NMDS+PERMANOVA Analysis

The NMDS algorithm was used to study the similarity or difference in the composition of Phytoplankton functional groups communities, and PERMANOVA (Adonis) was used for testing and identification. Nonparametric multivariate analysis of variance for sample distance (Bray–Curtis). To reveal the distribution differences in phytoplankton functional groups among different sampling sites, the NMDS (Non-metric Multidimensional Scaling) algorithm was employed for dimensionality reduction analysis of community compositions to visualise the similarities between samples. The significance of inter-group differences was tested using PERMANOVA with 999 permutations. For evaluating the stability of stress values, the reliability of results was verified through 200 repeated calculations [32].

#### 2.4.5. Prediction of New Random Forest Model

Corresponding analyses were conducted using the Random Forest extension package (rfPermute, R version 3.1) under R version 3.1. The R value was computed via the tidyverse package (R version 3.1), while plot exploration was carried out with the ggplot2 package (R version 3.1) in R. Errors were quantified using out-of-bag (OOB) data—this dataset was further used to determine the relative importance of each functional group involved in the analysis, and these groups were subsequently ranked based on the resulting importance values. The random forest model employed 1000 decision trees, with performance evaluated using 5-fold cross-validation (where the training set accounted for 80% and the validation set for 20% of the data). The feature splitting parameter mtry was set to the square root of the total number of features” to ensure the reproducibility and scientific rigour of the results [33].

#### 2.4.6. Study on the Model of Niche Width and Interspecific Relationship

Using R version 3.1, the spaa package was called for data analysis and plotting. The calculation formulas are as follows: Niche breadth (Bi) was measured using Levins’ index [34]. Niche overlap (Oik) was calculated with Pianka’s overlap index.

$$Bi=\frac{1}{\sum_{j=1}^{r}P_{ij}^{2}}$$

where Bi is the niche breadth of the i-th species, j represents the quadrat, and *r* denotes the number of quadrats.

$$Oik=1-\frac{1}{2}\sum_{j=1}^{r}\left|p_{ij}-p_{kj}\right|$$

where Oik is the niche overlap coefficient between species i and k, Pij and Pkj are the abundances of species i and k at sampling site j, respectively.

*Interspecific Association*

The variance ratio method (VR) was used to measure the overall (association), and the χ^2^ test was combined to qualitatively determine interspecific associations [35].

The overall association was calculated using the variance ratio method with the following formula:

*Variance of species relative abundance*:

$$\sigma_{T}^{2}=\sum_{i=1}^{a}p_{i}{\left(1-Ρi\right)}^{2}$$

*Variance of species richness*:

$$S_{T}^{2}=\frac{1}{N}\sum_{j=1}^{N}{\left(T_{j}-t\right)}^{2}$$

*Variance Ratio*:

$$VR=\frac{S_{T}^{2}}{\sigma_{T}^{2}}\;\;W=VR\cdot Ν$$

Formula Description: S: Total number of dominant zooplankton species; N: Number of sampling sites; ni: Number of sampling sites where species i occurs; T_j_: Number of dominant zooplankton species in sampling site j. If VR > 1: Overall positive association; If VR < 1: Overall negative association; If VR = 1: No interspecific association. If W < χ^2^_0.95_ (N) or W > χ^2^_0.05_ (N). Overall connectivity obvious (*p* < 0.05); χ^2^_0.95_(N) < W < x^2^_0.05_ (N). Overall association was not significant *(p* > 0.05).

Interspecific Association Test:

The stability of community structure and interspecific relationships can be reflected by interspecific associations, which play a positive role in the prediction of zooplankton succession [36]. In this study, the point correlation coefficient (Φ) was used to test and quantify the association and association strength between each pair of dominant species [36]:

$$pcc=\frac{ad-bc}{\left(a+b)(a+c)(b+d)(c+d\right)}$$

a: Number of sampling sites where both species are present; b: Number of sampling sites where species B is present but species A is absent; c: Number of sampling sites where species A is present but species B is absent; d: Number of sampling sites where neither species is present. The *PCC* (point correlation coefficient) ranges from [−1, 1].

## 3. Results

### 3.1. Composition of Functional Groups of Phytoplankton

**Figure 2:**
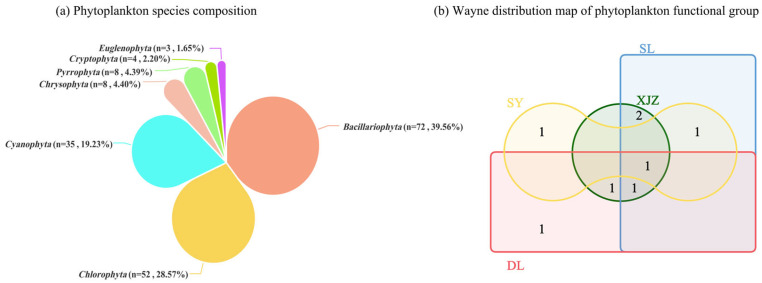
Venn diagram of phytoplankton species composition in four affiliated reservoirs of the Tarim River.

A total of 182 phytoplankton species belonging to 7 phyla were identified in 40 sampling sites during both the dry season and wet season (Figure 2a). The phytoplankton collected across four seasons from the affiliated reservoirs of the Tarim River Basin can be categorised into 18 functional groups, specifically A, D, E, F, H1, J, K, L0, M, MP, N, P, S1, SN, X1, X2, and Y. Among these, eight groups—A, D, H1, L0, M, MP, P, and S1—are the dominant functional groups. Among them, the dominant functional groups in the SY region are S1, A, and P; those in the DL region are S1, M, MP, and L0; those in the SL region are S1, A, D, L0, and H1; and those in the XJZ region are S1, M, D, L0, and H1. The common dominant functional group across the four reservoirs is S1. Functional group P is an exclusive dominant functional group in the SY region, and MP is an exclusive dominant functional group in the DL region, with significant differences in species composition among regions (Figure 2b).

**Figure 3:**
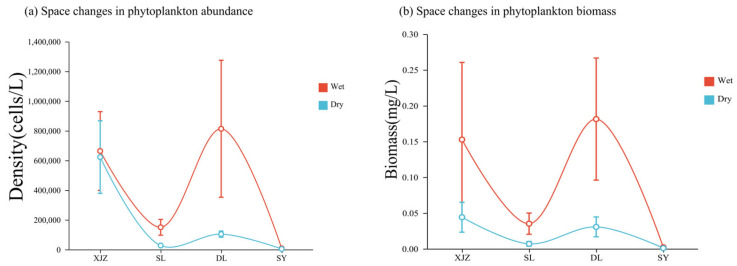
Spatial-temporal variation diagram of phytoplankton richness and biomass in four affiliated reservoirs of the Tarim River.

Figure 3a illustrates the spatiotemporal dynamic characteristics of phytoplankton cell abundance. From a temporal perspective, the seasonal fluctuation amplitude of phytoplankton in the XJZ Reservoir is relatively small; in contrast, the seasonal variations in other reservoirs are characterised by a much greater amplitude of abundance change in the wet season than in the dry season, with the DL Reservoir showing the most prominent difference in abundance between the wet and dry seasons. In terms of spatial distribution, the DL Reservoir exhibits the largest spatial amplitude of abundance, while the spatial amplitudes of abundance in the SL and XJZ Reservoirs are relatively close. As a water source, the SY Reservoir, due to its unique geographical condition of being perennially stocked by the Tarim River, has a spatiotemporal amplitude of abundance far lower than that of the other three reservoirs.

Figure 3b reveals the spatiotemporal dynamic pattern of phytoplankton biomass. Temporal sequence analysis shows that the fluctuation range of biomass in the SY Reservoir between the wet season and dry season is relatively narrow; in contrast, the biomass fluctuation amplitude of the other three reservoirs in the wet season is significantly higher than that in the dry season, a trend that is basically consistent with the seasonal variation in phytoplankton abundance. Notably, although the seasonal difference in phytoplankton abundance in the XJZ Reservoir is not significant, its biomass fluctuates drastically, indicating that the phytoplankton community structure in this reservoir has undergone seasonal succession. In terms of horizontal spatial distribution, the biomass fluctuation amplitude of the DL Reservoir is significantly higher than that of the other three reservoirs, indicating a broader distribution characteristic. In contrast, the spatial variability of biomass in the SY Reservoir is significantly lower, with a relatively narrow distribution range.

**Figure 4:**
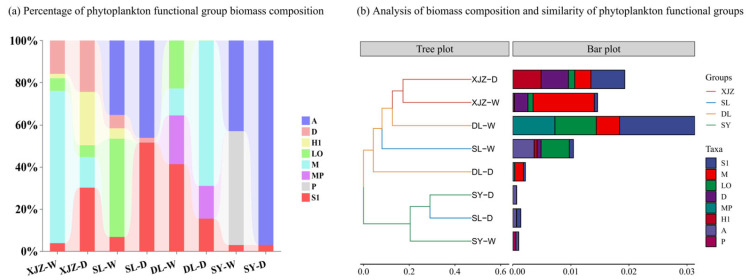
Biological composition and percentage distribution of phytoplankton functional groups for phytoplankton in four affiliated reservoirs of the Tarim River.

**Figure 5:**
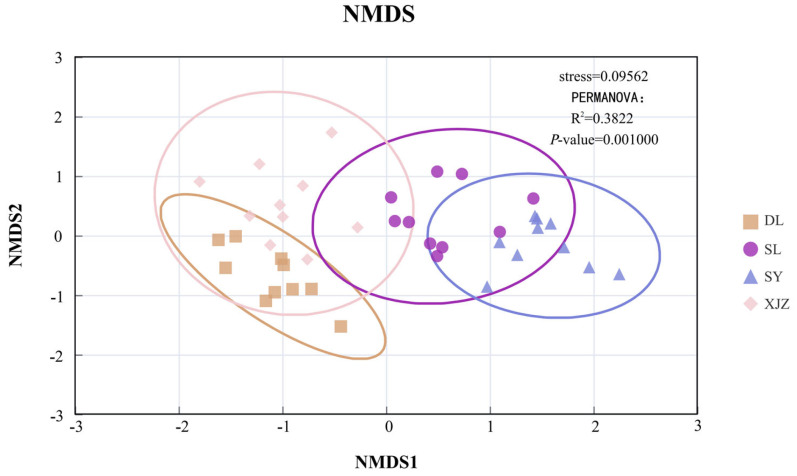
Distribution of phytoplankton NMDS in four affiliated reservoirs of the Tarim River.

The phytoplankton collected across four seasons from the affiliated reservoirs of the Tarim River Basin can be classified into 18 functional groups, namely A, D, E, F, H1, J, K, L0, M, MP, N, P, S1, SN, X1, X2, and Y. Based on the FG (functional group) classification method, functional groups with a relative biomass exceeding 5% are defined as dominant functional groups. In this study, there are eight dominant functional groups in total, including A, D, H1, L0, M, MP, P, and S1 (Table 1). Figure 4a illustrates the spatial and temporal distribution of phytoplankton functional groups. The variation in phytoplankton functional groups across spatiotemporal scales is evident. In the XJZ Reservoir, functional group M dominates during the wet season, accounting for up to 72% of the total, while its proportion gradually decreases to just 14% in the dry season, showing a distinct seasonal variation. In the SL region, the proportion of functional group L0, which accounted for 47% in the wet season, dropped to 0% in the dry season, indicating a dramatic shift in the community. In the DL region, functional group S1, which dominated in the wet season, was replaced by functional group M as the dominant group. In the SY region, the relatively even distribution of functional groups P and A shifted to a dominance of group A in the dry season. Overall, the species composition of functional groups has changed significantly, and the aquatic environment exhibits different trophic types across seasons. The similarity of phytoplankton functional community compositions across different reservoirs was analysed using the Bray–Curtis similarity coefficient (Figure 4b). Results indicate that, among the four reservoirs, the biomass of phytoplankton functional groups exhibits considerable differences—with no consistent distribution pattern observed across the study sites with the biomass in DL being significantly higher than that in other reservoirs. The composition of phytoplankton functional groups in SL and SY shows the highest similarity during the dry season. The similarity between the wet season in SY and the dry seasons in SY and SL is the next highest. On the whole, there are considerable differences in species composition across the four regions. To fully examine the clustering relationships among the four reservoirs, NMDS (Non-metric Multidimensional Scaling) clustering analysis was performed in this study, and the results are presented in Figure 5. From the comprehensive analysis, it can be seen that DL and XJZ share relatively high similarity, with SY and SL following closely behind. while the similarity between DL and XJZ is low. The overall difference is significant (*p* < 0.01).

### 3.2. Analysis of Spatiotemporal Dynamics of Water-Body Environmental Factors

**Figure 6:**
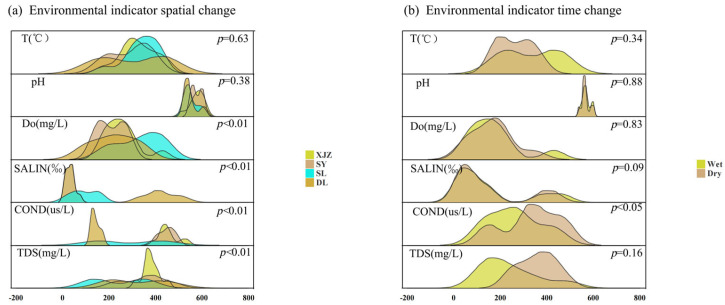
Spatiotemporal Difference Analysis of Physical and Chemical Indicators in the Water Bodies of Four Affiliated Reservoirs of the Tarim River. Note: A is spatial heterogeneity; B is temporal heterogeneity.

The spatial variations in water environmental factors are presented in Figure 6a. Results from the analysis of variance (ANOVA) reveal that all environmental factors, with the exception of temperature and pH, display distinct spatial differences. Specifically, DO in the XJZ reservoir is significantly higher than that in the other three reservoirs; the SY reservoir exhibits notably higher SALIN compared to the other three, suggesting a tendency toward saline-alkali water, while also having the lowest COND; and the SL reservoir has the highest TDS content. The seasonal variations in water environmental factors are illustrated in Figure 6b. In terms of seasonal patterns, pH, DO, and SAL show a high degree of overlap in the aquatic environment and maintain a relatively stable seasonal trend. COND and TDS are significantly higher during the dry season than in the wet season, whereas the temperature is markedly lower in the dry season. Overall, the water environmental factors show seasonal variation.

**Figure 7:**
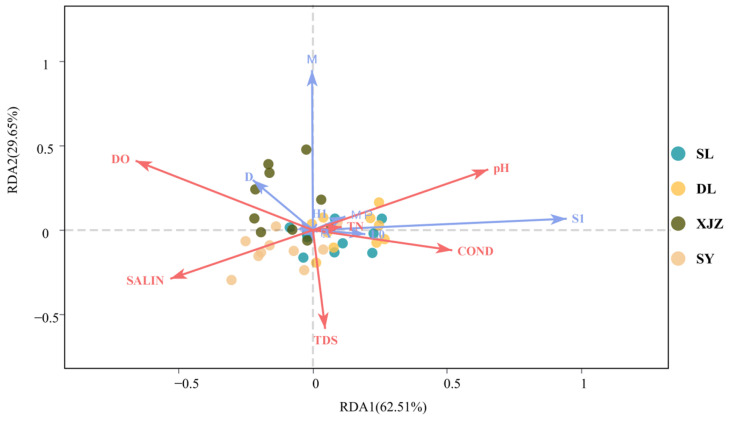
RDA of Phytoplankton functional groups in four reservoirs along Tarim River Basin and Associated Environmental Variables.

**Figure 8:**
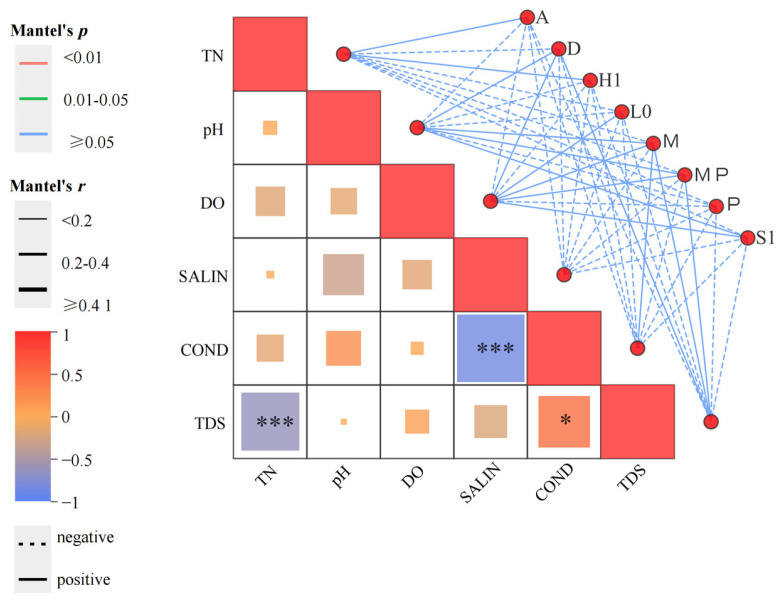
Phytoplankton and environmental factors Mantel test heat-map plot. Note: The line width represents the magnitude of the absolute Mantel correlation coefficient (|Mantel’s *r*|); the line colour corresponds to the range of the significance *p*-value (Mantel’s *p*); and the line type (solid or dashed) indicates the sign of the correlation coefficient. Specifically, * denotes *p* < 0.05, *** denotes *p* < 0.001, while the absence of an asterisk indicates *p* > 0.05.

DCA (Detrended Correspondence Analysis) was performed using Canoco for Windows 4.5 software, and the SD (scaling factor) value was found to be greater than 3. Therefore, RDA (Redundancy Analysis) was applied to explore the effects of environmental factors on phytoplankton. The explanatory rate of RDA1 was 62.51%, that of RDA2 was 29.65%, and the cumulative explanatory rate of RDA1 and RDA2 reached 92.16%. On the RDA1 axis, pH, COND, and TDS exhibited positive correlations, whereas all other environmental factors exhibited negative correlations. On the RDA2 axis, DO and pH presented positive correlations, with all other environmental factors showing negative correlations. Most environmental factors had no significant correlation with the biomass of phytoplankton functional groups. In contrast, the correlations among phytoplankton functional groups were more significant (Figure 7). This figure demonstrates the significance of environmental factors and dominant large benthic communities. On the right side, the network applies the Mantel test to analyse the correlation between individual environmental factors and species. Specifically, line width represents the absolute value of the correlation coefficient (Mantel’s *r*), line colour corresponds to the range of the significance *p*-value (Mantel’s *p*), and line type (solid or dashed) indicates the sign of the correlation coefficient (Figure 8).

### 3.3. Quantitative Evaluation of the Functional Group Niche Contribution Model

**Figure 9:**
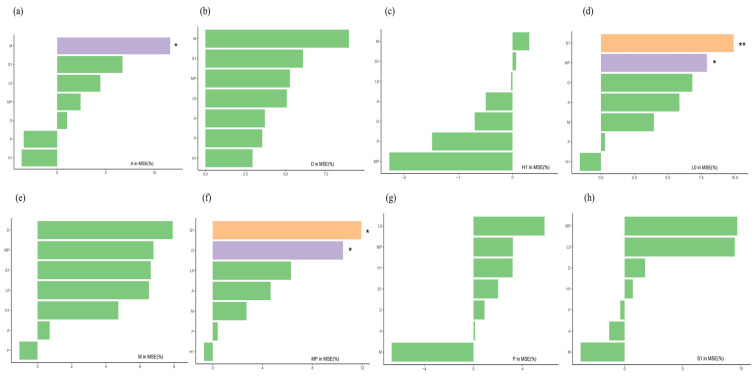
Prediction of the importance of the mutual influences among phytoplankton functional groups in four affiliated reservoirs of the Tarim River based on the random forest model. Note: (**a**): The significance of functional groups M, S1, L0, MP, D, P, and H1 to the A functional group; (**b**): The significance of functional groups M, S1, L0, MP, A, P, and H1 to the D functional group; (**c**): The significance of functional groups M, S1, L0, MP, A, P, and D to the H1 functional group; (**d**): The significance of functional groups M, S1, H1, MP, A, P, and D to the L0 functional group; (**e**): The significance of functional groups L0, S1, H1, MP, A, P, and D to the M functional group; (**f**): The significance of functional groups L0, S1, H1, M, A, P, and D to the MP functional group; (**g**): The significance of functional groups L0, S1, H1, M, A, MP, and D to the P functional group; (**h**): The significance of functional groups L0, P, H1, M, A, MP, and D to the S1 functional group; * denotes *p* < 0.05, ** denotes *p* < 0.01.

R version 3.1 was used, with the novel random forest package (rfPermute) invoked for the corresponding analysis. The tidyverse package was applied to calculate the r-value, and the ggplot2 package was used for plotting exploration. Errors were calculated using out-of-bag (OOB) data, and the relative importance of each functional group involved in the analysis was generated and ranked. The random forest model analysis revealed that the strongest influencing factor on functional group A is M, followed by S1 and L0, while P and H1 show negative correlations. The main influencing factor on D is M, followed by S1 and MP, with all other factors exhibiting positive correlations. H1 is positively correlated only with M and S1, and negatively correlated with all others. L0 shows a highly significant positive correlation with S1 (*p* < 0.01) and a significant correlation with MP (*p* < 0.05); it is negatively correlated only with S1 and positively correlated with all other factors. The main influencing factor on functional group M is functional group D, followed by MP and S1; among other factors, only P is positively correlated, while all others are negatively correlated. MP shows a highly significant difference from S1 (*p* < 0.01) and a significant positive correlation with D (*p* < 0.05); it is negatively correlated only with functional group H1. Functional group P is negatively correlated only with M, and positively correlated with all other factors. S1 has a strong positive correlation with MP and L0, followed by D and H1; it is negatively correlated with all other factors (Figure 9).

**Figure 10:**
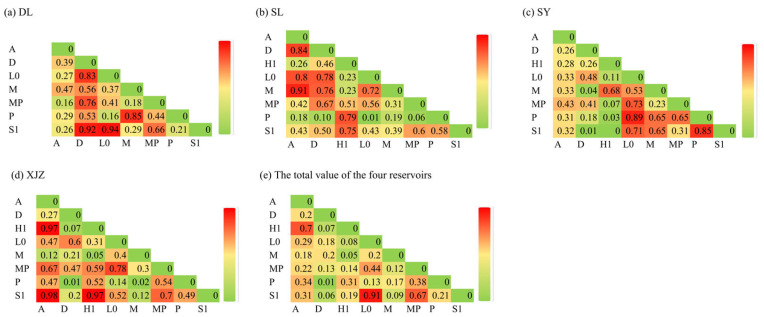
Ecological niche overlap of phytoplankton functional groups in four subsidiary reservoirs in Tarim River Basin. Note: (**a**) (DL), (**b**) (SL), (**c**) (SY), (**d**) (XJZ), (**e**) (Total value of four reservoirs).

The niche overlap values of phytoplankton functional groups in the affiliated reservoirs of the Tarim River Basin are shown in Figure 10. The range of niche overlap across the four reservoirs is 0.00–0.98. Specifically, the niche overlap range in DL is 0.16–0.94, with five pairs exhibiting high overlap rates (Oik > 0.7). Among these, the niche overlap between L0 and S1 is the highest, while the niche overlaps between A & MP and between P & L0 are the lowest. In SL, the niche overlap range is 0.01–0.91, with eight pairs having high overlap rates (Oik > 0.7). The niche overlap between M and A is the highest here, and that between P and L0 is the lowest. For SY, the niche overlap range is 0–0.89, with four pairs showing high overlap rates (Oik > 0.7). The niche overlap between P and L0 is the highest in SY, while that between S1 and H1 is the lowest. In XJZ, the niche overlap range is 0.01–0.98, with five pairs having high overlap rates (Oik > 0.7). The niche overlap between A and S1 is the highest in XJZ, and that between P and D is the lowest. Across all four reservoirs, the niche overlap range is 0.00–0.98, with two pairs exhibiting high overlap rates (Oik > 0.7). The niche overlap between S1 and L0 is the highest overall, while that between P and D is the lowest.

**Figure 11:**
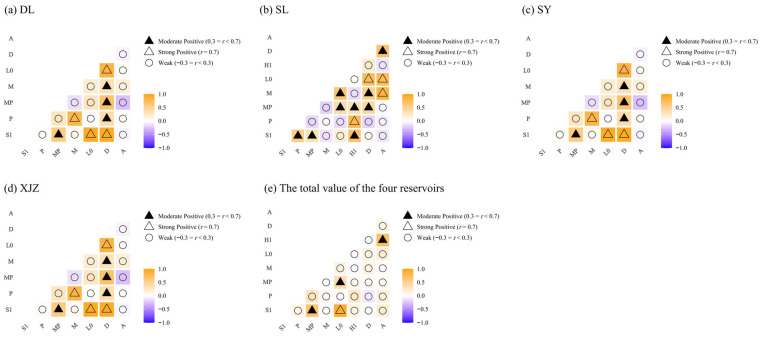
Pearson’s correlation analysis under different fertilisation gradients. Note: (**a**) (DL), (**b**) (SL), (**c**) (SY), (**d**) (XJZ), (**e**) (Total value of four reservoirs).

According to the results of the Pearson correlation coefficient test, 16 pairs of positive correlations and five pairs of negative correlations were found in DL, among which four pairs were significantly positive (*r* > 0.7). In SL, 17 pairs of positive correlations and 11 pairs of negative correlations were identified, with four pairs being significantly positive (*r* > 0.7). For SY, 13 pairs of positive correlations and 15 pairs of negative correlations were detected, including two pairs of significantly positive correlations (*r* > 0.7). In XJZ, 18 pairs of positive correlations and 10 pairs of negative correlations were found, with three pairs showing significant positive correlations (*r* > 0.7). Regarding the interrelationships among phytoplankton functional groups across all four reservoirs, 24 pairs of positive correlations and four pairs of negative correlations were observed, with one pair of significantly positive correlations (*r* > 0.7) (Figure 11). The correlation analysis results indicate that, except for SY, the number of negative correlations in other reservoirs is less than that of positive correlations. The correlation relationships across the four reservoirs are more intricate, with positive correlations far outnumbering negative ones. The upstream area, as a drinking water source with long-term water exchange, may be the main factor leading to more negative correlations than positive ones.

**Figure 12:**
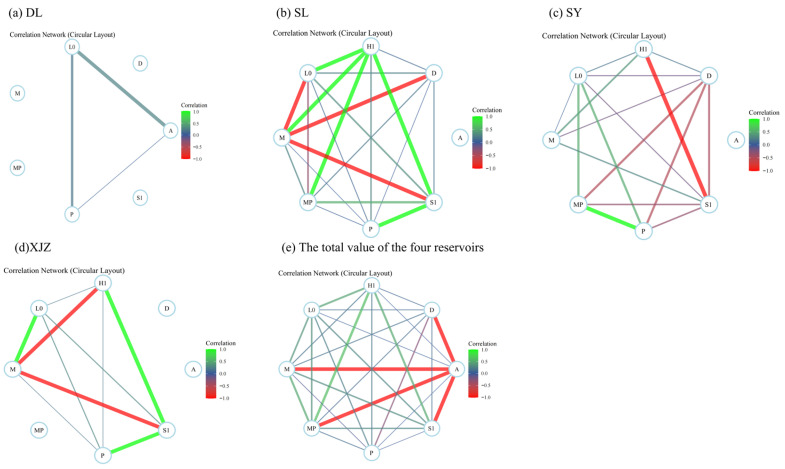
Species association diagram of four subsidiary reservoirs and total value of four reservoirs in Tarim River Basin. Note: (**a**) (DL), (**b**) (SL), (**c**) (SY), (**d**) (XJZ), (**e**) (Total value of four reservoirs).

According to the network diagram showing interspecific relationships among zooplankton functional groups in different estuarine areas (Figure 12), three pairs showed positive correlations in DL; 17 pairs showed positive correlations and four pairs showed negative correlations in SL; 8 pairs showed positive correlations and nine pairs showed significant negative correlations in SY; and eight pairs showed positive correlations and two pairs showed negative correlations in XJZ. The network diagram of phytoplankton functional groups across the four reservoirs revealed 21 pairs of positive correlations and seven pairs of negative correlations.

## 4. Discussion

### 4.1. Changes in the Structure of Phytoplankton Functional Groups in Arid Regions

Specific phytoplankton groups, as biological indicators of the aquatic habitat status, exhibit corresponding changes in their distribution patterns with the variation in aquatic environmental conditions [37]. In addition, phytoplankton communities possess the ability to adapt to environmental changes through natural succession processes, thereby ensuring the stability of aquatic ecosystems to a certain extent [38]. In this study, a total of 182 phytoplankton species belonging to 7 phyla were identified from 40 sampling sites across four affiliated reservoirs in the Tarim River Basin during both the dry and wet seasons. The phytoplankton collected across four seasons were classified into 18 functional groups, among which eight groups (A, D, H1, L0, M, MP, P, and S1) were dominant. Phytoplankton functional groups classify taxa with the same adaptability and habitat requirements, which can more accurately reveal the dynamic relationships between the functional characteristics of phytoplankton communities and the aquatic environment [39]. The dominant functional groups are mostly from Bacillariophyta, which is consistent with the research results of Min Wenwu et al. [40] on the Weihe River. *Pseudanabaena limnetica* in the dominant functional group S1 is characterised by heat resistance, light tolerance, and nitrogen fixation [41,42] and the high nitrogen concentration in the reservoir is conducive to its proliferation.

In the SL area, functional group L0, which accounted for a relatively high proportion in the wet season, disappears in the dry season, leading to a dramatic change in the community structure. In the DL arAt the spatiotemporal scale, the changes in phytoplankton functional groups are significant. In the XJZ area, functional group M dominates during the wet season, while its proportion gradually decreases in the dry season, showing a pronounced seasonal variation. This is consistent with the results mentioned by Wenqi Gao in the phytoplankton research in the Yangtze River [43]. functional group S1, which was dominant in the wet season, is replaced by functional group M as the dominant group in the dry season. In the SY area, the relatively even distribution of functional groups P and A shifts to a complete dominance of group A in the dry season. Phytoplankton groups exhibit significant changes in functional groups both in the same area during different periods and in different areas during the same period. The findings of this study are consistent with those of Mutshinda [44] in the research on phytoplankton functional groups. It indicates that substantial changes in community composition are closely related to the aquatic environment’s different trophic types in various seasons [43]. The dynamic change sequence of phytoplankton is mainly determined by the interaction of thermal stability, nutrient concentration, and the morphological and physiological adaptation characteristics of phytoplankton (such as suspension mechanisms, nutrient absorption and utilisation efficiency, light-capturing ability, carbon fixation rate.). These factors collectively constitute the selection mechanism for the occurrence or disappearance of phytoplankton in specific habitats [45,46]. This further indicates that the significant change in SL area, where L0, the most dominant functional group in the wet season, no longer remains dominant in the dry season, is closely related to aquatic environmental factors.

The dominant functional groups in reservoirs basically reflect the trophic level of the reservoir. In this study, except for the SY reservoir, the other three reservoirs all have the S1 functional group as the most dominant one. Functional group S1 primarily inhabits eutrophic waters, and its dominance is common in many water body studies, both domestically and internationally. For example, in the Hungarian rivers [47], shallow lakes in Hungary [48], Xionghe Reservoir in Hubei Province [49], as well as Henggang Reservoir, Shuilianshan Reservoir and Nanping Reservoir in Guangdong Province during the wet season, the S1 functional group is dominant [50]. The auxiliary reservoirs in the Tarim River Basin are located in a unique geographical location with minimal human disturbance. Their hydrological conditions are closely linked to the Tarim River, and the frequent fluctuations in reservoir water levels directly lead to significant variations in water depth. Notably, the main runoff of the Tarim River discharges into Reservoir SY—a hydrological characteristic that not only results in a much lower phytoplankton abundance in Reservoir SY compared to other reservoirs in the basin but also causes a significant divergence in its phytoplankton species composition from the others as shown in Table 2. This finding is consistent with the research results of the Valeriano-Riveros et al. team, who observed changes in phytoplankton composition during water level fluctuations in plateau tropical reservoirs in central Mexico. Their study showed that during the high-water period, phytoplankton not only exhibited higher taxonomic diversity but also underwent significant changes in biomass, while their abundance during the low-water period was significantly lower than that during the high-water period [51]. This is consistent with the results of the present study.

### 4.2. Interactions Among Phytoplankton Functional Groups and Their Relationships with Aquatic Environmental Factors in Arid Regions

In recent years, research on the interspecific interactions of phytoplankton has gradually expanded; however, studies on phytoplankton in high-latitude arid regions are still in their infancy [53,54]. The spatial niche breadth of a species reflects its ability to distribute and utilise resources [55]. Populations with a broad niche exhibit strong adaptability to the environment and possess considerable niche breadth. As their population density increases, interspecific competition intensifies accordingly. Competitive behaviours among phytoplankton species are also one of the important factors affecting population changes [56]. Functional groups occupying different niches may have competitive relationships, leading to mutual influence between them and jointly acting on the dynamic changes in community structure [57]. The bottom-up effects of light, nutrients, and temperature, the top-down effect of fish predation, and interspecific competition are all significant factors affecting the growth of phytoplankton, and they also influence the distribution of phytoplankton functional groups [58].

Plankton plays a key role in regulating the balance of aquatic ecosystems. As a critical environmental factor affecting phytoplankton growth, temperature in this study showed a significant negative correlation with functional group A. The dominant species in functional group A is *Cyclotella* sp., and appropriate growth conditions can accelerate the growth of *Cyclotella* sp., this is consistent with the findings of Saros in his ecological study on Cyclotella [59]. Bergmann’s Rule is applicable to this phytoplankton community [60], which represents a previously established regularity. However, this study conducted in-depth verification using Redundancy Analysis (RDA) and reached a new conclusion: temperature is not the primary factor driving changes in the community composition. Instead, pH, COND, DO, and other factors have a greater impact on phytoplankton functional groups, with pH being a key driving factor. Within a specific range, an increase in water pH leads to weakly alkaline aquatic environments, which accelerate the absorption of CO2 from the air, promote photosynthesis in aquatic plants, and facilitate the absorption of dissolved inorganic carbon by phytoplankton [61,62].

To obtain the predicted values of importance indices for phytoplankton functional group interactions, a random forest model was utilised in this research. The most important positive factors for the interspecific relationships of phytoplankton functional groups in the affiliated reservoirs of the Tarim River were identified as M, MP, L0, and S1. Model prediction analysis revealed that when the abundance or functional effect of the S1 functional group is significantly enhanced, the corresponding ecosystem will exhibit dynamic characteristics of rapid growth of phytoplankton dominated by *Navicula* sp and *Chroococcus* sp. These phytoplankton (such as those in L0 and MP) exhibit strong resistance to interference and have a broad niche. The results of this study are consistent with those of Liao [63] in his/her research on phytoplankton in canyon reservoirs. With the increase in S1, resource availability becomes sufficient, which reduces interspecific competition. Consequently, the available resources for *Navicula* sp., *Chroococcus* sp., and other species increase, leading to a concurrent rise in their abundances [64]. When the abundance of H1 increases to a certain level, all phytoplankton functional groups except M and S1 are inhibited and enter a competitive relationship. The dominant species in functional group H1 is *Anabaena* sp. *Anabaena* sp. can maintain large-scale biomass production over a long period in a small eutrophic lake. Eutrophic lakes are characterised by causing photo-oxidative death in many other phytoplankton species [65]. By enhancing the synthesis of carotenoids, *Anabaena* sp. can protect chlorophyll a from photo-oxidation and prevent it from catalysing other intracellular photo-oxidative reactions. Therefore, the random forest model predicts that when the H1 functional group increases significantly, the abundances of other functional groups will be inhibited, which is related to the survival strategy of *Anabaena* sp.

### 4.3. Niche Overlap Characteristics of Phytoplankton Functional Groups in Arid Regions

Niche overlap reflects the relationship between resource utilisation and the spatial distribution of two or more phytoplankton species in the same habitat. Also, it indicates the competitive relationship among species for resource utilisation [66]. Algae with a considerable niche breadth can affect the community structure of algal communities and the status of the aquatic environment [67]. Therefore, monitoring the status of algae with a considerable niche breadth can help determine the essential changes in reservoir water quality. This study investigated the interspecific niche overlap of dominant functional groups in different reservoirs. In general, there was a certain degree of niche overlap in reservoirs during different periods, indicating that the phytoplankton community was generally in a stable state without obvious habitat specialisation. In terms of the significance rate of niche overlap values, the order is SL > DL > XJZ > SY, indicating that the similarity in ecological characteristics and resource utilisation among dominant species in DL is the highest. However, niche overlap does not necessarily lead to competition; competition arises only when resources are scarce and there is a high degree of niche overlap [66]. Further refined research on phytoplankton niche in Nanhai Lake of Baotou revealed significant seasonal variations in the niche breadth and niche overlap of dominant phytoplankton species. Specifically, both the niche breadth and niche overlap values of dominant phytoplankton species during the ice-covered period were lower than those during the non-ice-covered period, which indicates more intense competition for environmental resources among species in the non-ice-covered period [67,68]. This ecological result is common in the ecosystem of arid regions. Species with a larger niche breadth also have a higher probability of niche overlap with other species.

In the DL Reservoir, functional group D shows a high overlap rate with all other functional groups. Dominated by *Synedra* sp., functional group D is adapted to nutrient-rich waters with low transparency. It is characterised by tolerance to low oxygen and low temperatures, and belongs to a broad-niche species, thus exhibiting a high degree of niche overlap with other functional groups [69]. All four reservoirs harbour highly overlapping phytoplankton functional groups. However, this study found that in the broader environment, the overall overlap rate of phytoplankton functional groups decreased significantly; only functional groups S1 and L0 exhibited a high overlap rate, while the overlap rates of other functional groups were low. In the broader environment, interspecific competition among phytoplankton decreased due to increased space and nutrients. Only functional group S1, with a relatively large niche breadth, can exist in waters ranging from oligotrophic to eutrophic and in medium to large water bodies, thus exhibiting high overlap with the mesotrophic functional group L0. This is consistent with Lei’s results in the XG Reservoir during summer [70].

### 4.4. Overall Association and Interspecific Association of Dominant Phytoplankton Functional Groups in Arid Regions

Overall, interspecific association can reflect the community succession process and the stability of species composition [71]. The results of the overall association of dominant phytoplankton functional groups in the affiliated reservoirs of the Tarim River indicate that the phytoplankton communities in the DL, SL, and XJZ reservoirs generally tend to be stable. Under conditions of little change in environmental factors, the community structure is unlikely to undergo significant fluctuations. Although there is a specific association in the phytoplankton community structure of the SY Reservoir, stable and coordinated relationships have not been formed among species to achieve optimal resource utilisation. The community as a whole is unstable, prone to fluctuations or even degradation, and exhibits an independent distribution pattern characterised by contingency and randomness [72]. Interspecific association refers to the mutual spatial distribution between two species, revealing interspecific interactions and community dynamics from a qualitative perspective [73]. Interspecific correlation, on the other hand, reveals the linear relationship between two species and the degree of probability of their co-occurrence from a quantitative perspective [74]. The test results of interspecific association and interspecific correlation show both consistencies and differences; combining the two can more comprehensively explain interspecific relationships.

By comparing the results, this study found that the Chi-square test and the Pearson correlation test are consistent with each other. Both analytical methods showed that interspecific positive associations are dominant in DL, SL, and XJZ reservoirs, while negative associations are dominant in the SY Reservoir. Generally, for communities in the early stage of succession, the degree of interspecific association is low, the ratio of positive to negative associations is small, and the community is in an unstable stage [75]. With the succession and renewal of communities, interspecific associations gradually become positive, community stability increases, and communities develop successively towards the climax stage [76]. That is, the SY Reservoir is in the initial stage of succession, while the communities in DL, SL, and XJZ reservoirs are generally stable. As a drinking water source, the SY Reservoir is subject to intensive human disturbance. In the specific context of alpine lake regions, landscape characteristics—including altitude, bedrock properties, vegetation, hydrological networks, lake morphology, and other related factors—can narrow and influence the adjacent physical and chemical conditions as well as resource availability. Meanwhile, phytoplankton may be subjected to biological constraints such as predation and interactions with zooplankton [77]. Moreover, as a reservoir for replenishing water to the Tarim River, significant water level fluctuations in the reservoir area lead to long-term changes in phytoplankton communities. These results are consistent with those of interspecific correlation analyses, indicating that the community is in the initial stage of succession with an unstable structure.

In ecological research, interspecific association and correlation are key concepts that accurately reflect the complex network of interactions within biological communities during specific periods. Generally, species pairs with positive association tend to exhibit interspecies mutualism and complementarity; in contrast, species pairs with negative association are more inclined to show interspecific exclusion and competitive behaviours [78]. Beyond the top-down inhibitory effect of grazing, zooplankton also exert a bottom-up promotional effect on phytoplankton through nutrient excretion; the relative intensity of these two effects determines the final regulatory outcome. This study confirms that the bottom-up effect induced by zooplankton nutrient excretion actually outweighs the top-down inhibitory effect from their grazing behaviour—a mechanism that also explains the synchronous increase in the biomass of zooplankton and phytoplankton observed in some water bodies [79]. Based on the research results of AC interspecific association and niche overlap values, this study found that the niche overlap values of these species all exceed 0.7, which indicates that the stronger the positive association between species, the greater the similarity in resource utilisation and the possibility of niche overlap, and the higher the tendency toward co-survival and joint resistance to harsh environments among species. As a lake in the cold and arid region, a study on the phytoplankton community in Wuliangsuhai Lake employed the Association Coefficient (AC) and variance ratio method to quantify the interspecific associations among dominant species. The community exhibited an overall positive association; however, the presence of a large number of species pairs with weak associations quantified the characteristic of relative independence among species in the community [80]. Similarly, a study conducted on Xiashan Reservoir in Shandong Province confirmed, by means of indicators such as the association coefficient, that 76% of the species pairs showed significantly positive associations [81].

By comparing the two methods, this study also found specific differences in their results. The reason may lie in the fact that the Pearson correlation coefficient test is a parametric test method, which requires species to follow a continuous normal distribution. However, most species in nature exhibit a clumped distribution, thereby leading to certain limitations in the results of the Pearson correlation coefficient test. In contrast, interspecific correlation, based on abundance data between species pairs, can provide a basis for analysing the pattern of species rise and fall in the community. The same viewpoint has also been proposed by Hong Run Tu in the article titled Interspecific associations of the main tree populations of the Cyclobalanopsis glauca community in Karst hills of Guilin, Southwest China [82]. The interspecific relationships it indicates are consistent with those revealed by interspecific association, but there are differences in sensitivity. In interspecific association analysis, two species generally show a positive association when they co-occur or co-disappear in quadrats. However, from a quantitative perspective, they may exhibit a negative correlation if one species has a large quantity while the other has a small quantity [83]. Random forest models further confirmed that interspecific relationships are positively driven primarily by functional groups M, MP, L0, and S1. For S1 specifically, its increase enhances the growth of functional groups dominated by *Navicula* sp. and *Chroococcus* sp. through reducing resource competition. Conversely, the expansion of functional group H1 inhibits other groups; this effect is associated with its adaptive strategy of resisting photo-oxidation in eutrophic environments. Therefore, in this study, the results of the quantitative analysis method show more pairs with negative associations, which is consistent with the findings of Liu Yipeng et al. [84].

Furthermore, what interspecific association and correlation reveal is merely the outcomes or current status of interspecific and intraspecific competition, without elaborating on the ecological processes and mechanisms that lead to such outcomes, nor the relative contribution of each mechanism. Therefore, future studies should continue long-term monitoring of the affiliated reservoirs of the Tarim River and conduct in-depth exploration of the impacts of various ecological factors on community stability and their underlying mechanisms. This approach will help further elaborate on the ecological mechanisms governing the formation of interspecific associations.

## 5. Conclusions

This study investigated the community structure of four representative reservoirs in the Tarim River Basin, Xinjiang, China, and their relationships with environmental factors. From 2023 to 2024, seasonal sampling of phytoplankton functional groups was conducted in four reservoirs in the upper reaches of the Tarim River, with a total of 18 functional groups identified, 8 of which are dominant functional groups. Redundancy Analysis (RDA) revealed that water pH, electrical COND, and dissolved DO are the key environmental factors influencing the distribution of phytoplankton functional groups; their variations can directly regulate the dominance and standing stock of different functional groups. The phytoplankton communities in DL, SL, and XJZ are dominated by positive associations, reflecting that the community structures of these three reservoirs are relatively stable and less prone to drastic fluctuations under stable environmental conditions. In contrast, SY is dominated by negative associations, indicating that the phytoplankton community in this reservoir is in the early stage of succession with poor stability. This phenomenon is closely related to the environmental characteristics of SY Reservoir, which is subject to strong human disturbances and undertakes the task of replenishing water to the Tarim River, resulting in significant water level fluctuations.

Random forest models further revealed that four functional groups (M, MP, L0, and S1) are the main positive drivers of interspecific relationships. Among them, the S1 functional group can promote the growth of functional groups dominated by *Navicula* sp. and *Chroococcus* sp. by reducing resource competition. In contrast, the expansion of the H1 functional group inhibits other functional groups, which is associated with its adaptive strategy of resisting photooxidation in eutrophic environments.

## Figures and Tables

**Table 1:** Representative algal genera and habitat characteristics of phytoplankton functional groups.

Functional Group	Representative Species	Ecological Characteristics
A *	*Cyclotella* sp.	Poor nutrition, cleanliness, deep water
D *	*Synedra* sp. *Nitzschia* sp.	Contains nutrients and low transparency
E	*Dinobryon* sp.	Poor or heterotrophic, small, shallow
F	*Oocystis* sp.	mesotrophic-eutrophic, clear-water lake
H1 *	*Anabaena* sp.	Rich in nutrients, stratified, low in nitrogen, shallow water
J	*Scenedesmus* sp. *Actinastrum* sp.	High nutrition, mixed, shallow water
K	*Aphanocapsa* sp.	Rich in nutrients, shallow waterRich in nutrients, shallow water
LO *	*Merismopedia* sp. *Chroococcus* sp.	Poor to eutrophic, medium to large water bodies
M *	*Microcystis* sp.	Small to medium, eutrophic to hypereutrophic
MP *	*Navicula* sp. *Surirella* sp.	Constant agitation, muddy water, shallow water
P *	*Melosira* sp. *Closterium* sp.	Medium nutrient, shallow water, thermocline
S1 *	*Limnothrix* sp. *Planktothrix* sp.	Medium richness, mixed turbidity, low transparency
SN	*Raphidiopsis* sp.	Warm, Mixed
X1	*Chlorella* sp. *Ankistrodesmus* sp.	Overfertile, shallow water
X2	*Chlamydomonas* sp.	Rich in nutrients, shallow water
Y	*Cryptomonas* sp. *Cryptomonas ovata*	High nutrition, mixed, shallow water

Note: In this study, the designated functional groups were indicated with *.

**Table 2:** Basics of Reservoir 2024 [52].

Reservoir	Reservoir Capacity (10,000 m^3^)	Water-Level Fluctuation (m)	Water Storage Purpose
DL	7376.33	0.4	Agricultural irrigation
SL	6233.63	0.2	Agricultural irrigation
SY	10345.36	1.2	Drinking water source, Ecological water supply
XJZ	5678.33	0.4	Agricultural irrigation

Note: The value is the average of the four seasons.

## Data Availability

The data supporting this study’s findings are available from the corresponding authors upon reasonable request.
